# Anatomic Risk Factors Predisposing Female Handball Players to Non-contact Anterior Cruciate Ligament Injury

**DOI:** 10.7759/cureus.104522

**Published:** 2026-03-01

**Authors:** Zacharoula Papadopoulou, Angelo V Vasiliadis, Eleni Maria Vrampa, Nikiforos Galanis

**Affiliations:** 1 Department of Competitive Sports, Division of Team Handball, School of Physical Education and Sports Science, Aristotle University of Thessaloniki, Thessaloniki, GRC; 2 Department of Physical Education and Sports Science at Serres, Aristotle University of Thessaloniki, Thessaloniki, GRC; 3 Department of Physical Education and Sports Science, Aristotle University of Thessaloniki, Thessaloniki, GRC; 4 1st Orthopaedic Department, George Papanikolaou General Hospital, Aristotle University of Thessaloniki, Thessaloniki, GRC

**Keywords:** anatomic risk factor, anterior cruciate ligament injury, exercise, female athlete, handball

## Abstract

Handball involves high-intensity movements that increase the risk of anterior cruciate ligament (ACL) injury, making the identification of anatomical risk factors essential. Female handball players are more vulnerable to non-contact ACL injury than male and key contributors include coronal and sagittal knee alignment abnormalities, such as increased Q-angle, valgus alignment and knee hyperextension, which predispose players to the valgus-collapse mechanism frequently observed during cutting and landing. Bony morphology in females, including wider pelvis, narrow intercondylar notch width, altered femoral anteversion and steeper posterior tibial slopes, further elevates injury risk by reducing joint constraint and increasing anterior tibial translation. Intrinsic ligament characteristics, such as smaller anterior cruciate ligament size and greater joint laxity, also heighten susceptibility in female athletes. Anterior cruciate ligament injury risk is therefore multifactorial, arising from the interaction of alignment, bony geometry and ligament properties. Understanding these factors supports targeted prevention and more effective screening in female handball athletes.

## Editorial

Handball is a high-intensity sport that places significant physical demands on athletes. Specific aspects of the sport, such as quick pivoting, side-cutting maneuver, landing from jumps and forceful acceleration or deceleration, predispose players to serious knee injuries, including anterior cruciate ligament (ACL) tears [1]. ACL injury remains a catastrophic injury for athletes, with females being especially affected, making essential to understand the anatomical risk factors to tailor prevention strategies [2]. Since 1990, the incidence of ACL injury has been reported to be highest among elite female players, with 0.82 ACL injuries per 1000 playing hours compared with 0.31 injuries per 1000 playing hours in males [3]. This gender difference has been consistently confirmed in subsequent studies [3,4]. In this context, the question “Which anatomical factors place female handball players at greater risk?” becomes highly relevant.

Knee alignment as a risk factor

**Figure 1:**
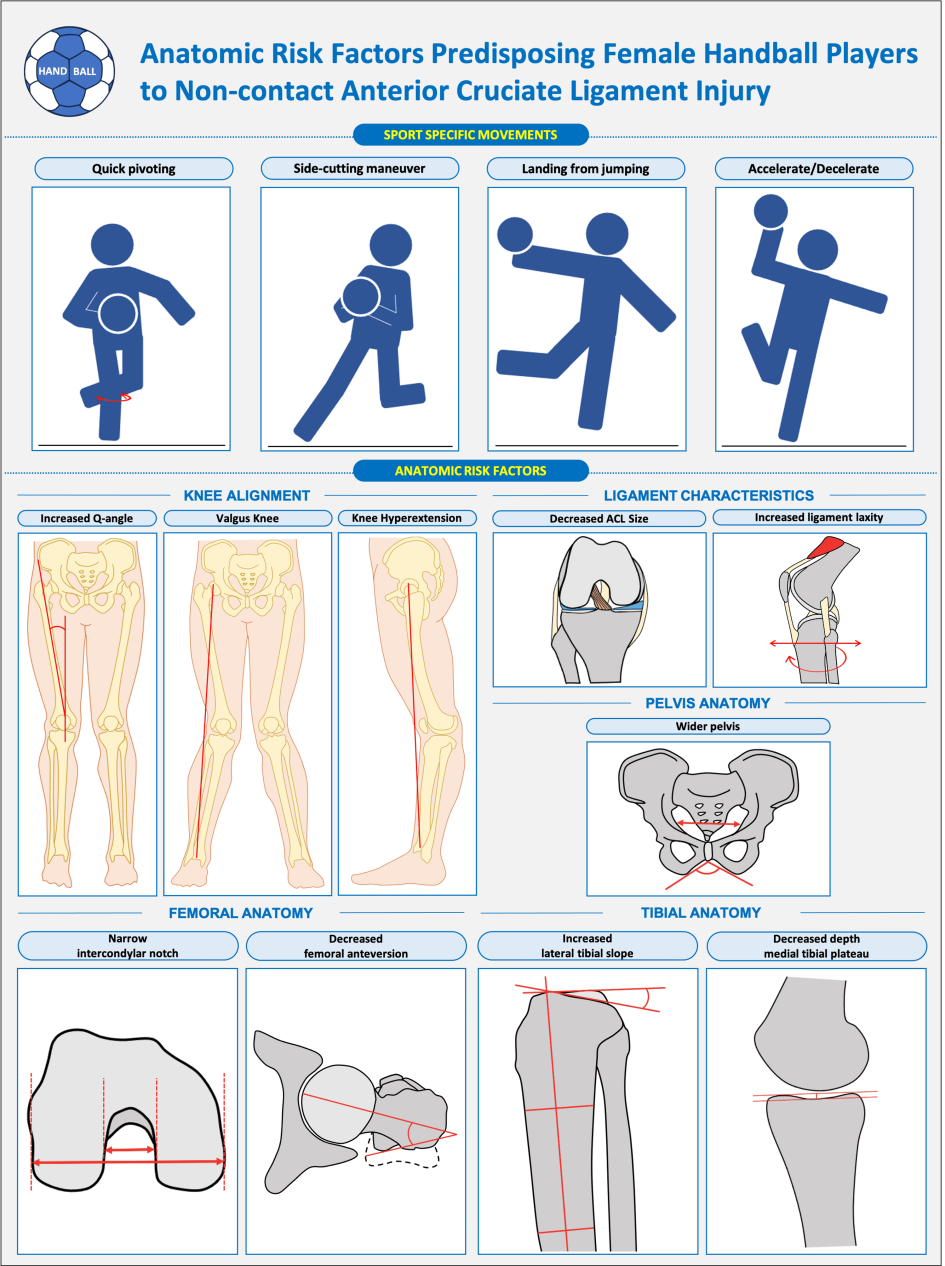
Anatomic risk factors predisposing female handball players to non-contact anterior cruciate ligament injury The figure was created using Microsoft PowerPoint by the author Angelo V. Vasiliadis (AVV).

The quadriceps angle, commonly referred to as the Q-angle, is defined as the angle between the line extending from the anterior superior iliac spine to the center of the patella and the line from the patella to the tibial tuberosity [5]. It serves as a straightforward measure of static knee alignment and is typically larger in females than in males. A wider pelvis, more commonly seen on average in females, can shift the reference point outward, thereby increasing the Q-angle, while a shorter femur may promote leg eversion, also contributing to a larger Q-angle. An increased Q-angle has been associated with a higher risk of knee injuries, including ACL tears [5]. Supporting this, Kamatsuki et al. reported that valgus knee alignment was associated with an increased risk for non-contact ACL injury among elite female handball and soccer players [2]. They also observed that knee hyperextension increased the likelihood of secondary ACL injury (Figure 1). Previous studies using video analysis of ACL injuries indicate that, in female handball players, most ACL injuries occur during rapid directional changes, such as when passing an opponent, or following one-leg landing from jumps [6,7]. In these situations, the injury mechanism typically involves a forceful valgus collapse near full knee extension, combined with slight rotation of the tibia [7]. Together, these findings emphasize the significance of frontal and sagittal knee alignment in the injury pathway.

Bony morphology as a risk factor

Beyond knee alignment, other anatomical factors, such as femoral intercondylar notch width, femoral anteversion, tibial plateau slope and pelvic anatomy, have also been explored (Figure 1) [1,8,9]. A case-control study of female handball players found that those with a narrow femoral notch were six times more likely to sustain an ACL injury compared to players with a wider notch width [9]. The authors further suggested that an anterior opening of less than 17 mm should be considered a risk factor for ligament injury, particularly in athletes who continue to participate in high-level handball. Similarly, Nakase et al., in a prospective cohort study of female high school basketball and handball players, found that decreased femoral anteversion was associated with an increased risk of non-contact ACL injury [1]. In contrast to this finding, other literature has reported that a 1° increase in hip anteversion corresponds to a 1.78-fold increase in the probability of ACL injury [10]. Differences in measurement methods may explain the discrepancy, highlighting the need for further research to clarify these findings.

The tibial plateau slope is another critical anatomical factor linked to ACL injury risk. A systematic review and meta-analysis found that steeper posterior, medial and lateral tibial slopes significantly increase the risk of ACL injury [8]. In this context, it has been demonstrated that a 10° increase in posterior tibial slope can result in a 6 mm increase in anterior tibial translation, thereby tripling the load on the ACL [11]. Moreover, the geometry of the tibial plateau, particularly the depth of the medial concavity in combination with the posterior tibial slope, plays a key role in knee stability. A shallower medial tibial depth and steeper posterior slopes reduce joint constraint, thereby increasing susceptibility to ACL injury [12].

In addition to femoral and tibial morphology, pelvic anatomy has also been proposed as an intrinsic risk factor influencing knee biomechanics and thus predisposition to ACL injury (Figure 1). A wider pelvis, which is more common in females, tends to increase the Q-angle, a configuration that may encourage dynamic knee valgus and increased medial stress on the knee during landing, cutting or pivoting maneuvers in a handball game [1,5,13]. In support of this, a recent review summarizing gender-based anatomical differences identified a wider pelvis, in combination with a larger Q-angle and steeper tibial slopes, among the set of morphological characteristics that may contribute to the elevated ACL injury risk in female athletes [14].

Ligament properties as a risk factor

Intrinsic ligament properties also influence susceptibility to ACL injury (Figure 1). Literature shows that females generally exhibit greater ligament laxity than males, partly due to hormonal influences, particularly the effects of estrogen, which decreases collagen synthesis and increases ligament laxity [15]. Significant increases in ACL laxity have been reported during both the follicular and luteal phases of the menstrual cycle, suggesting that hormonal surges in estrogen and progesterone may contribute to greater ligamentous laxity and potentially elevate ACL injury risk during these phases [15].

In addition to laxity, the size of the ACL itself has been identified as a potential anatomical risk factor [2, 12, 16]. Individuals who have sustained a non-contact ACL injury often exhibit a significantly smaller ACL diameter in their contralateral, uninjured knee compared to matched controls [16]. This finding suggests that a smaller ACL possesses reduced structural strength, thereby increasing its vulnerability to rupture [2,16]. Furthermore, Gupta et al. reported that a combination of a narrow intercondylar notch width and a low ACL volume was associated with a higher risk of ACL injury among athletes participating in non-contact sports [16]. Collectively, these findings indicate that decreased ACL size may represent an underlying anatomical predisposition to non-contact ACL injury.

Knee joint laxity, both anteroposterior and rotational, has also been investigated as a contributing risk factor [1,2]. Increased laxity can arise from generalized joint hypermobility or from anatomical features such as a steeper tibial slope, which generates greater anterior shear forces and enhanced quadriceps loading, thereby increasing sagittal knee laxity [2,8]. Excessive knee extension and genu recurvatum, which exceed normal physiological limits of ligamentous laxity, have been linked to a higher incidence of ACL injury [12,17].

While anatomical features alone do not determine whether a female handball player will sustain an ACL injury, they represent important indicators of individual susceptibility. No single factor is sufficient to cause an ACL tear; rather, it is the interaction and combination of multiple anatomical characteristics that create a predisposition to injury. Recognizing these structural risk factors enables coaches, clinicians and sports scientists to design more targeted prevention and screening strategies. The key challenge moving forward lies in integrating anatomical assessment with dynamic movement analysis and load management to more effectively mitigate injury risk and enhance player safety.
